# Different clusters of *Candidatus* ‘Methanoperedens nitroreducens’-like archaea as revealed by high-throughput sequencing with new primers

**DOI:** 10.1038/s41598-018-24974-z

**Published:** 2018-05-16

**Authors:** Sai Xu, Chen Cai, Jianhua Guo, Wenjing Lu, Zhiguo Yuan, Shihu Hu

**Affiliations:** 1 Advanced Water Management Centre, The University of Queensland, Brisbane, 4072 Australia; 2 School of Environment, Tsinghua University, Beijing, 100084 China; 3 Key Laboratory for Solid Waste Management and Environment Safety (Tsinghua University), Ministry of Education of China, Tsinghua University, Beijing, 100084 China

## Abstract

The newly discovered *Candidatus* ‘Methanoperedens nitroreducens’ (*M*. *nitroreducens*), mediating nitrate-dependent anaerobic oxidation of methane, is an important microorganism in linking carbon and nitrogen cycles. In order to explore the diversity of *M*. *nitroreducens*-like archaea in various environmental niches with advanced high-throughput sequencing, new primers based on alpha subunit of methyl-coenzyme M reductase gene were designed. The PCR results demonstrated that the new primers could effectively detect *M*. *nitroreducens*-like archaea from an enrichment culture dominated by *M*. *nitroreducens* as well as samples collected from a natural freshwater lake and a full-scale wastewater treatment plant (WWTP). By high-throughput sequencing, more than 30,000 *M*. *nitroreducens*-like sequences were obtained. Phylogenetic analysis of these sequences along with published sequences showed that *M*. *nitroreducens*-like archaea could be divided into three sub-branches (named as Group A, Group B and Group C in this study). Clear geographical difference was observed, with Group A and Group B dominating samples in Queensland (Australia) and in European ecosystems, respectively. Further quantitative PCR revealed that the *M*. *nitroreducens*-like archaea were more abundant in WWTP than the freshwater lake. The study provided a large number of sequences for *M*. *nitroreducens*-like archaeal communities, thus expanded our understanding on the ecological diversity of *M*. *nitroreducens*-like archaea.

## Introduction

Methane is an important greenhouse gas with global warming potential 28 times than that of carbon dioxide and contributes up to about 20% of global warming forces^1^. The methane concentration in atmosphere has been increasing at 1% annually since the industrial revolution^2^, thus attracting great attention from both scientists and governments.

Microbial methane oxidation is an effective way to reduce the potential methane emission into the atmosphere^3^. Aerobic oxidation of methane and anaerobic oxidation of methane are the two major pathways for microbial methane oxidation^4^. Anaerobic oxidation of methane (AOM) can be coupled with various electron acceptors including sulfate^5^, nitrate^6^/nitrite^7^ and metal oxides^8,9^. With the exception of AOM coupled with nitrite reduction, which was mediated by *Candidatus* “Methylomirabilis oxyfera” belonging to the NC10 bacterial phylum^7,10^, all other known anaerobic methane oxidation reactions were mediated by anaerobic methanotrophic archaea (ANME). To date, different clusters of ANMEs coupled with sulfate reduction (including ANME-1, ANME-2a/b, ANME-2c and ANME-3) have been ubiquitously found in various marine ecosystems^5,11–15^ and some extreme environments (e.g. volcanoes)^16,17^. These ANMEs were believed to consume up to 90% methane generated in marine (21) via the reverse methanogenesis pathway, with the first step catalysed by methyl-coenzyme M reductase (MCR). The *mcr*A gene encoding the alpha subunit of MCR has evolved as the key marker gene for studying the diversity of ANMEs^4^.

Recently, a new cluster of ANMEs, ANME-2d, was found in freshwater system. It has been successfully enriched in a bioreactor fed with methane, nitrate and ammonium^6^. Unlike other clusters of ANMEs discussed above, the enriched ANME-2d coupled AOM to nitrate reduction and was named as *Candidatus* ‘Methanoperedens nitroreducens’ (*M*. *nitroreducens*). In the phylogenetic tree, *M*. *nitroreducens* was clustered within the family of *Methanoperedenaceae*. Metagenomic analysis revealed that the *M*. *nitroreducens*, like other ANMEs, harboured all the essential genes encoding the reverse methanogenesis pathway^6,18,19^.

In order to detect the *M*. *nitroreducens*-like archaea from different environmental niches, specific primers targeting 16 S rRNA and *mcr*A genes have been designed recently^20,21^. Using these primers, *M*. *nitroreducens*-like archaea were detected in wastewater treatment plant (WWTP), river sediment, channel sediment, paddy soil and sea sediment^20,21^. In these studies, specific primers were used for polymerase chain reaction (PCR) and the PCR products were then cloned before the Sanger sequencing. With the recent development of high-throughput sequencing technology, the PCR product can be sequenced directly without cloning and much more data can be obtained in less time compared with Sanger sequencing. Thus, the high-throughput sequencing technology is considered to be more convenient for microbial community profiling^22^. However, the PCR product using the current *mcr*A gene primers was 1191 bp^20^, and was not suitable for the current high-throughput sequencing technology, which required the PCR product to be less than 500 bp^23^. Thus, new primers suitable for high-throughput sequencing are necessary to better evaluate the distribution and diversity of *M*. *nitroreducens*-like archaea.

In this study, new PCR primers targeting the *mcr*A gene were designed to realize the high-throughput sequencing with the PCR products. Based on our enrichment culture with *M*. *nitroreducens* as the dominant microorganism^6^, PCR reactions were conducted to evaluate the validity of primers and the reaction conditions were then optimized. The primers were further tested with samples collected from a freshwater lake (natural ecosystem) and a WWTP (engineered ecosystem). This study intends to provide a new molecular tool for high-throughput detection of *M*. *nitroreducens*-like archaea and expands the sequences database for ecological investigation of *M*. *nitroreducens*-like archaea.

## Results

### Primer design and evaluation

Firstly, this study evaluated if the available specific primers for *M*. *nitroreducens*-like archaea (Table S1) were applicable with the DNA extracted from enriched *M*. *nitroreducens* cultures before the new primers were designed. The primers combinations, DP142F/DP779R in the first round and DP142F/DP569R in the second round^21^, were not able to produce positive results. *M*. *nitroreducens*-like sequences was successfully amplified with the primers McrA169F and McrA1360R although the fragment (1191 bp) is too long to be sequenced with the high-throughput sequencing technology (Figure S1).

**Figure 1:**
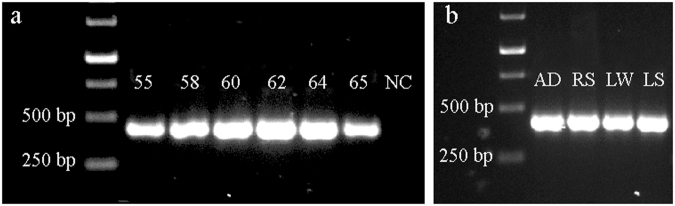
Gel image of the PCR products from (**a**) EC and (**b**) engineered (AD, RS) and freshwater (LW, LS) samples. The numbers in (**a**) indicated the different annealing temperatures (°C). NC meant negative control. The full-length gels were presented in Supplementary Figure S2 and Figure S4.

When using the new nested PCR strategy developed in this study, a dominant and bright PCR band at the expected size (363 bp) was identified, while no amplification was shown in the negative control sample (NC) (Fig. 1a and Figure S2). The band intensity analysis showed only one peak existed in each gel lane, indicating only one band was amplified for each sample (Figure S3). The PCR amplification efficiency was relatively high when the annealing temperature was between 60–64 °C. *M*. *nitroreducens*-like archaea from WWTP and freshwater lake samples were also successfully identified by the new nested PCR method (Fig. 1b and Figure S4). Thus, our results confirmed that the new strategy, using the primers McrA169F and McrA1360R in the first-round PCR and then the new primers McrA997F and McrA1360R in the second-round PCR, was highly efficient for amplification of *M*. *nitroreducens*-like archaea from environmental niches.

### Phylogenetic analysis

The PCR products generated by the new nested PCR method from WWTP and freshwater lake samples were sequenced on the Miseq platform. In total, 55,972 raw sequences were obtained. Most of the sequences (>99%) obtained were similar to the *mcr*A gene in the *M*. *nitroreducens* genome LKCM01000102. Dissimilarity sequences (<80%) were discarded (less than 10 sequences per sample) and normalization of the sequence number was conducted by extracting 7,542 sequences from each sample for fair comparison in the following analysis. The rarefaction curves showed that the number of newly detected OTUs grew slowly when the number of sequences was more than 5,000 without reaching the saturation point (Figure S5). These sequences were enough to cover *M*. *nitroreducens*-like archaeal communities.

**Figure 2:**
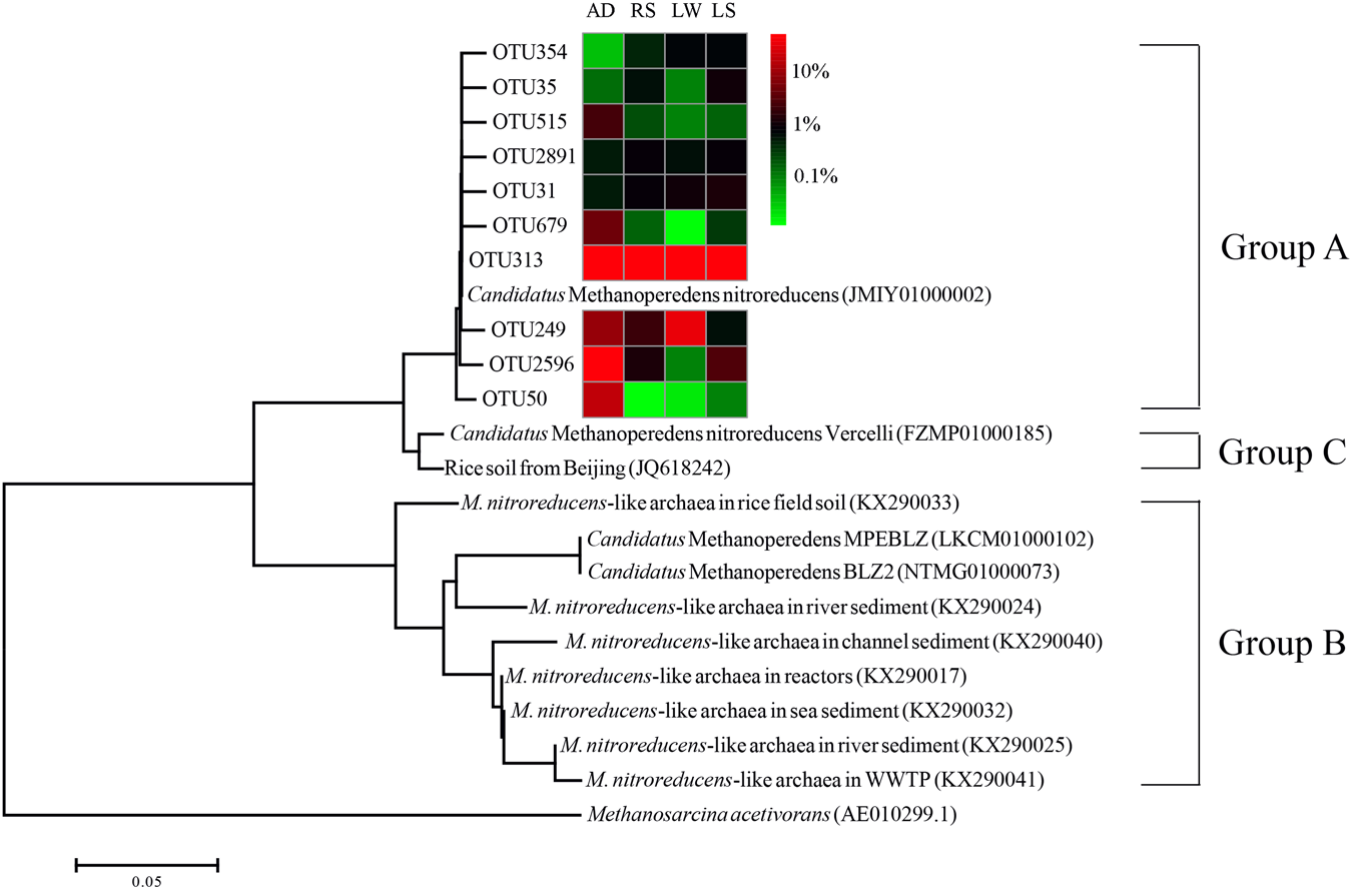
Phylogenetic tree showing the top 10 OTUs from the WWTP (AD, RS) and freshwater lake (LW, LS) samples. *Methanosarcina acetivorans* was used as the out-group. The scale bar represented 5% divergence. The abundance of the top 10 OTUs in each sample was shown in the heatmap referred to the colour key. AD: anaerobic digestion sample, RS: return sludge sample, LW: lake water sample, LS: lake sediment sample.

After deducing DNA sequences to protein sequences, 970 OTUs in total and 434–538 OTUs for each sample were obtained at the 0.03 cut-off level. The top 10 OTUs, accounting for more than 70% of the total sequences, were selected for phylogenetic analysis. All of the selected sequences had high similarity (>99%) to the *M*. *nitroreducens* genome LKCM01000102, medium similarity (~96%) to the *M*. *nitroreducens* genome FZMP01000185 and low similarity (<85%) to the *M*. *nitroreducens* genomes JMIY01000002 and NTMG01000073 (Table S2). The OTU313 was the most abundant OTU in all the samples and accounted for 63% of the total sequences. In the phylogenetic tree (Fig. 2), this OTU exhibited closer evolution relationship with the *M*. *nitroreducens* genomes LKCM01000102 (100% identity) and FZMP01000185 (97% identity) than other *M*. *nitroreducens* genomes JMIY01000002 and NTMG01000073 (both 83% identity) (Table S2). The phylogenetic tree also showed that these 10 OTUs were grouped into one cluster and was distinct from other sequences that have been reported elsewhere^19,20,24^. Based on the phylogenetic distance, three distinct groups of *M*. *nitroreducens*-like archaea were classified, which were named as Group A, Group B and Group C, respectively. Group A was mainly found in this study and our previously study^6^ while Group B and Group C has been reported elsewhere^18,19,24^.

### Diversity analysis

The alpha diversity indices were calculated to show the internal diversity of the samples. The Shannon index indicated that the *M*. *nitroreducens*-like archaea were more diverse in lake sediment than lake water sample (Table 1). For the WWTP samples (AD and RS), the diversity indices were similar. The values of sequences coverage were higher than 0.96, indicating that the sequencing covered a significant amount of the richness of *M*. *nitroreducens*-like archaea in these samples.

**Table 1:** Diversity indices of *M*. *nitroreducens*-like archaea in different samples.

Sample	OTU number	Shannon index	Simpson index	Chao index	Goodscoverage
AD	475	2.54	0.34	771	0.97
RS	471	2.34	0.46	820	0.97
LW	434	2.19	0.45	740	0.97
LS	538	2.77	0.36	947	0.96

### Quantitative analysis of *M. nitroreducens*-like archaea abundance

The newly designed primers McrA 997F and McrA 1360R were used to quantify the *M*. *nitroreducens*-like archaeal abundance. The calibration curve was generated by five orders of magnitude using the plasmid containing partial cloned *mcr*A gene. The calibration curve had a significant linear correlation (R^2^ = 0.9997) (Figure S6) and only one peak at 83 °C appeared in the melting curve (Figure S7). The amplification efficiency was almost 100% matching the theoretical calculation, confirming no unspecific amplification and therefore the results were highly reliable.

**Figure 3:**
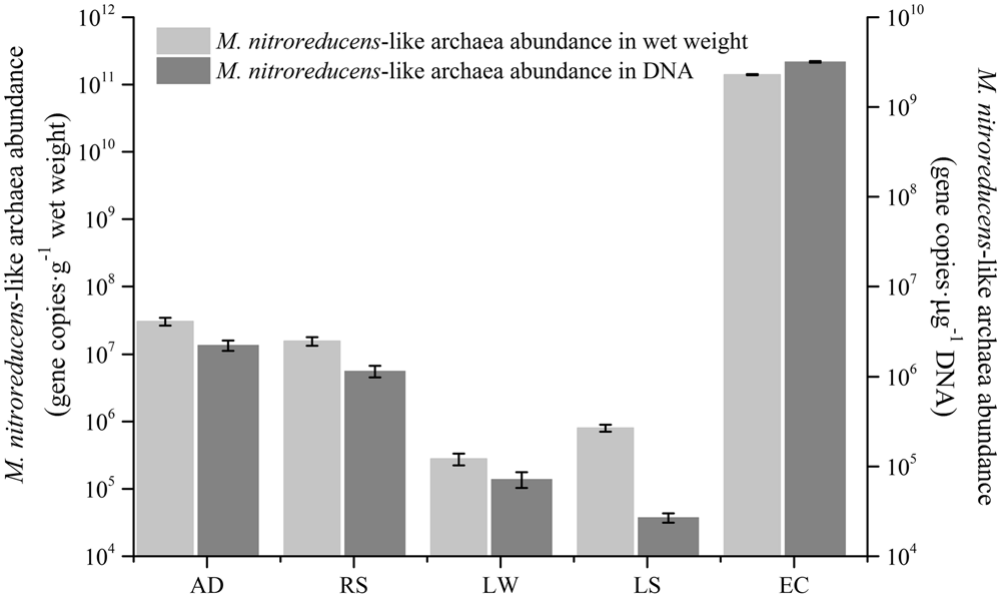
Quantitative PCR results showing *M*. *nitroreducens*-like archaea abundance in each sample. The values were shown as average ± standard deviation based on three independent replicates. The values in light and dark colour columns were calculated based on the wet weight and DNA concentration of samples, respectively. AD: anaerobic digestion sample, RS: return sludge sample, LW: lake water sample, LS: lake sediment sample, EC: enrichment culture.

The quantitative PCR results showed that *M*. *nitroreducens*-like archaeal abundance in natural and engineered ecosystems ranged from 2.79 × 10^5^ to 3.07 × 10^7^ copies per gram, equalling to 2.69 × 10^4^ to 2.24 × 10^6^ copies per µg DNA (Fig. 3). This result also suggested that the population density of *M*. *nitroreducens*-like archaea was relatively high in WWTP and low in freshwater lake. The results indicated the *M*. *nitroreducens*-like archaea was highly enriched in the bioreactor (EC) with the abundance 4–6 orders of magnitude higher than that in the other samples.

## Discussion

The diversity and distribution of *M*. *nitroreducens*-like archaea in ecosystems has attracted substantial attention recently due to its potential significance in reducing methane emission into atmosphere. Culture-independent PCR is a widely used molecular method to detect microorganisms from different environmental niches. Considering that very limited primers based on functional gene *mcr*A are available in literature^20^, this study aimed to develop a specific PCR method for the high-throughput detection of *M*. *nitroreducens*-like archaea in ecosystems.

Mining the public database (NCBI), four genomes of *M*. *nitroreducens* and 28 partial *mcr*A sequences of *M*. *nitroreducens*-like archaea were retrieved. Based on alignment of these sequences, new primers were designed and nested PCR method was introduced to realize high-throughput detection of *M*. *nitroreducens*-like archaea. The PCR results showed that single bright bands were visible at the annealing temperature ranges 55–65 °C and the amplification efficiency was relatively high when the annealing temperatures at range 60–64 °C. Based on the high-throughput sequencing (Miseq) results, more than 99% of the sequences (>30,000) obtained in this study showed high similarity (>99%) to the *M*. *nitroreducens* genome JMIY01000002, indicating the PCR method based on our designed primers were efficient to amplify *M*. *nitroreducens*-like archaea from different environmental niches.

We proposed to divide *M*. *nitroreducens*-like archaea into different sub-branches (Group A, Group B and Group C) based on the phylogenetic distance. All of the *M*. *nitroreducens*-like archaea related sequences detected in this study was clustered into Group A. Group B was previously reported from environment samples collected in Europe^20^ and enrichment culture with canal sediment^7,20^. Group C was newly reported enrichment cultures with paddy soil from Italy^19^ and it was of high similarity (98%) to another *mcr*A gene sequence that was also retrieved from paddy soil^24^. The difference of these groups is probably caused by different geographical condition. Some studies indicated geographical differences could lead to significant difference in microbial communities^22,25^. The annual temperature in our sampling site (northeast Australia) is higher than that in Europe and previous studies also indicated temperature could lead to the different clusters in ANMEs^26^.

The quantitative results showed that the abundances of *M*. *nitroreducens*-like archaea were higher in WWTP samples while relatively lower in natural lake samples. In a previous study, the McrA159F/McrA345R primers were used to quantify the *M*. *nitroreducens*-like archaea from different ecosystems^20^, which showed its abundance in sludge was about two orders of magnitude (10^5^ copies per gram wet weight) lower than that found in this study. Another study used the 16 s rRNA primers to detect the *M*. *nitroreducens*-like archaea in China, in which the abundance in lake sediment was similar to this study (10^4^ copies per µg DNA)^21^. The detection of *M*. *nitroreducens*-like archaea in various conditions indicated the wide distribution of this archaea in environments and it might play an important role in reducing the methane emission from environmental niches^27^.

In summary, this study provided a new powerful tool to discover *M*. *nitroreducens*-like archaea in natural and engineered environments. By using the high-throughput sequencing, a large number of *M*. *nitroreducens*-like archaea related sequences (>30,000) were added to the database, which significantly expanded the *M*. *nitroreducens*-like archaea sequences database (only 28 sequences available before this study). Based on these sequences, *M*. *nitroreducens*-like archaea were divided into three distinct groups. Recent study indicated *M*. *nitroreducens*-like archaea together with NC10 bacteria could play an important role in methane reduction in freshwater system^28^ but the detection method and distribution information of *M*. *nitroreducens*-like archaea is limited. With fast and specific molecular method for detection of *M*. *nitroreducens*-like provided by this study, its contribution in methane sink will be further discerned in various environments.

## Methods

### Sample collection

One *M*. *nitroreducens* enrichment sample, two engineered ecosystem samples and two freshwater samples were collected in this study. The 5 mL enrichment sample (EC) was collected from a lab-scale reactor that has been fed with nitrate and ammonium along with methane for more than 5 years^6^. The culture was dominated by *M*. *nitroreducens* (>40%) and anammox (~6%), facilitating a nitrate-nitrogen removal rate of 20–30 mg /L·d. The detailed operating conditions of the reactor can be found in literatures^29,30^.

The engineered ecosystem samples were collected from the anaerobic digester (AD) and the return sludge (RS) in the Luggage Point WWTP in Brisbane (27°22′ S/153°08′ E). The freshwater samples were collected from the lake located at the University of Queensland (27°29′ S/153°00′ E). The lake water (LW) and lake sediment (LS) samples were collected at three independent sites and were mixed together to get the representative samples. About 1 L of AD and RS samples, 50 mL LW sample and 20 g LS sample were collected, preserved in a cold container and quickly transferred to the cold room (4 °C) on ice. More details of the samples were listed in Table S3.

### Primer design

**Figure 4:**
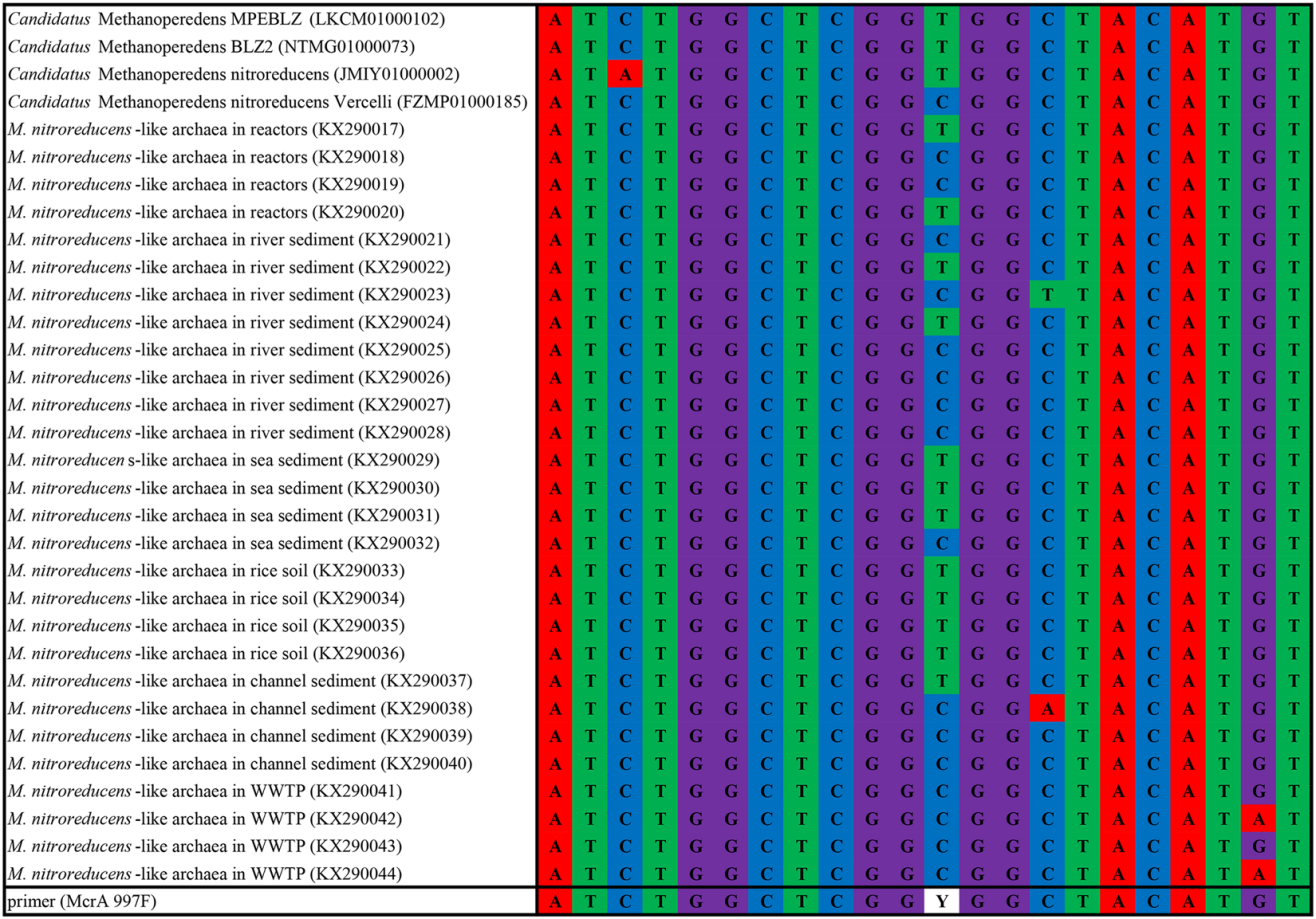
Alignment of primers with the related sequences downloaded from NCBI database.

The primers were designed based on the *mcr*A gene sequences of *M*. *nitroreducens* from the NCBI database. Four genomes of *M*. *nitroreducens* (LKCM01000102, JMIY01000002 and FZMP01000185) and 28 *mcr*A partial sequences of *M*. *nitroreducens*-like archaea (KX290017-KX290044) were downloaded. The alignment of these sequences was performed with Clustal X (version 2.0)^31^ and the conserved region was selected as the target for the primer design (Fig. 4). The primer design was then carried out using Primer Premier (version 6.0, Premier Biosoft International, USA). The McrA 997 F primer was newly designed in this study and the efficiency was confirmed in silico with the NCBI Primer-Blast tool.

### DNA extraction and PCR amplification

About 0.5 mL liquid samples or 0.4 g sediment samples were used for DNA extraction. The microbial DNA was extracted using the FastDNA SPIN Kit for Soil according to the manufacturer’s protocol (MP biomedicals, USA). The DNA quality and concentration were assayed using the Nanodrop-1000 spectrophotometer (Thermo Fisher Scientific, USA). The absorption ratio at 260/280 nm was in the required range of 1.8–2.0. All the extracted DNAs were stored at −20 °C for further analysis.

PCR reactions were carried out in a 25 µL mixture containing 12.5 µL premix (Thermo Fisher Scientific, USA), 10–20 ng DNA templates and 1 µL (20 µM) forward and reverse primers. Three PCR methods were tested to amplify the *M*. *nitroreducens*-like archaea from the EC sample. Firstly, the previously reported nested PCR method targeting the 16 s rRNA gene was used, in which DP142F/DP779R and DP142F/DP569R were used in the first and second rounds of amplification, respectively^21^. Secondly, the EC sample was directly amplified with McrA 169 F/McrA 1360 R primer^20^. Thirdly, a new nested PCR method was proposed. McrA 169 F/McrA 1360 R primer set was used in the first round of PCR, followed by the new primer combination McrA 997 F/McrA 1360 R in the second round. The thermal cycling was performed with an initial degeneration step for 3 min at 94 °C, followed by 35 cycles of denaturation for 30 s at 94 °C, annealing for 40 s, elongation for 40 s at 72 °C and the final elongation was 10 min at 72 °C. The annealing temperature was 55 °C for the DP142F/DP779R primers, 59 °C for the DP142F/DP569R primers, 57 °C for the McrA 169 F/McrA 1360 R primers and 55–65 °C for McrA 997 F/McrA 1360 R primers, respectively. In the nested PCR, the PCR products in the first round were diluted for 10 times with diethy pyrocarbonate treated water, which were then used as templates for the second round of PCR. Negative control (NC) was also performed using the DNA extracted from an aerobic mixed culture^32^. Detailed information of the primers was listed in Table S1.

### High-throughput sequencing and post-sequencing analysis

The sizes of the PCR products were verified by the agarose electrophoresis (1%). The correct size bands were cut off from gel, pooled together and then purified with QIAquick Gel Extraction kit (Qiagen, Germany).

The purified PCR products were then used for sequencing library preparation. Firstly, McrA 997 F/McrA 1360 R were modified to include the Illumina forward and reverse adapter sequences on their 5′ ends. The PCR reaction was performed using the modified primers and the condition was same as the mentioned above. Then the dual indices and Illumina sequencing adapters were attached to the PCR products using the Nextera XT i5 and i7 Index primer. This PCR reaction was performed with an initial denaturation at 95 °C for 3 min, followed by 10 cycles of 30 s at 95 °C, 45 s at 55 °C and 60 s at 72 °C, and the final elongation was 72 °C for 5 min^33^. The libraries were pooled in equimolar concentrations and high-throughput sequencing was performed on the Illumina Miseq platform (PE300) in Australian Centre for Ecogenomics.

Modified McrA 997 F primer (5′-3′):

TCGTCGGCAGCGTCAGATGTGTATAAGAGACAGATCTGGCTCGGYGGCTACATGT.

Modified McrA 1360 R primer (5′-3′):

GTCTCGTGGGCTCGGAGATGTGTATAAGAGACAGTGCCTCTTTGTGGAGGTACATGGA.

The sequencing data were analysed using the Mothur (version 1.33.2) according to the standard operating procedure^34,35^. Briefly, the sequences were sorted into different samples by matching the specific barcodes. Then, the primers, barcodes, adaptors and chimeras were trimmed off. The low quality sequences that contained ambiguous base or contaminants from 16 S rRNA gene were removed. The remaining DNA sequences were translated into the protein sequences and redundant sequences were removed. To fairly compare different samples at the same sequencing depth, normalization of the sequence number was conducted by extracting the same number of sequences from each sample for all the following analyses. The remaining protein sequences clustered into different OTUs using the UCLUST software (version 1.2.22q) at cut-off value of 0.03^36^. The representative sequence from each OTU was randomly selected and blasted with the *M*. *nitroreducens* genomic sequences LKCM01000102, JMIY01000002, FZMP01000185 and NTMG01000073 in the NCBI database. The sequences with similarity less than 80% were discarded.

### Phylogenetic analysis

The protein sequences were first aligned in Clustal X software and then the phylogenetic tree was built using the MEGA software (version 7.0.21)^37^ via the neighbour-joining method (1000 replicates). The previously reported *M*. *nitroreducens* genomic sequences^6,18,19,38^ and reference sequences from diverse environments (rice soil, sea sediment, channel sediment, river sediment and WWTP)^20^ were selected for phylogenetic analysis. *Methanosarcina acetivorans* (a typical methanogenic archaeon) was used as the out-group in the phylogenetic tree.

### Quantitative PCR

The abundance of *M*. *nitroreducens*-like archaea in each sample was quantified using the McrA 997 F/McrA 1360 R primers^20^. PCR products were ligated with pCR®2.1 vector and cloned into *Escherichia coli* competent cell using the TA Cloning Kit (Thermo Fisher Scientific, USA). Plasmid containing cloned *mcr*A gene was extracted and serial 1:10 dilutions of plasmid were used to generate calibration curve. The copy numbers in each sample were calculated based on comparison of the quantification cycle values with the calibration curve.

The quantitative PCR was conducted in a 10 μL mixture containing 5 μL SYBR Green Premix (Thermo Fisher Scientific, USA), 1~5 ng DNA templates, and 0.1 μL (20 μM) forward and reverse primers. An initial denaturing step of 95 °C for 10 min was followed by 40 cycles of 95 °C for 15 s and 60 °C for 1 min. The melting curve analysis was carried out after the amplification at temperatures ranged from 60 °C to 95 °C, increasing at a rate of 0.5 °C/s. Triplicate analyses were performed for each sample on the ViiA7 system (ABI Company, USA), and the negative control was also conducted simultaneously using the DNA free water as the template.

### Data availability

All of the raw sequencing data in this study have been deposited in the Sequence Read Archive database under accession numbers: SRR6058429-SRR6058432. Other data generated or analysed during this study are included in this manuscript and Supplementary materials.

## Electronic supplementary material

supplementary material
