# Supplementary material for: Different clusters of Candidatus ‘Methanoperedens nitroreducens’-like archaea as revealed by high-throughput sequencing with new primers
Source: Sci Rep. 2018 May 16;8:7695. doi: 10.1038/s41598-018-24974-z (PMC5955947; doi:10.1038/s41598-018-24974-z)
Supplement: Supplementary file 1 — supplementary material [file 41598_2018_24974_MOESM1_ESM.pdf]

Supplementary Materials

Different clusters of *Candidatus* 'Methanoperedens nitroreducens'-like archaea as revealed by high-throughput sequencing with new primers

Sai Xu [a,b,c], Chen Cai [a], Jianhua Guo [a], Wenjing Lu [b,c,*], Zhiguo Yuan [a,b], Shihu Hu [a,*]

[a] Advanced Water Management Centre, The University of Queensland, Brisbane 4072, Australia

[b] School of Environment, Tsinghua University, Beijing 100084, China

[c] Key Laboratory for Solid Waste Management and Environment Safety (Tsinghua University), Ministry of Education of China, Tsinghua University, Beijing 100084, China

[*] Correspondence to Shihu Hu (Email: s.hu@awmc.uq.edu.au) and Wenjing Lu (Email: luwenjing@tsinghua.edu.cn)

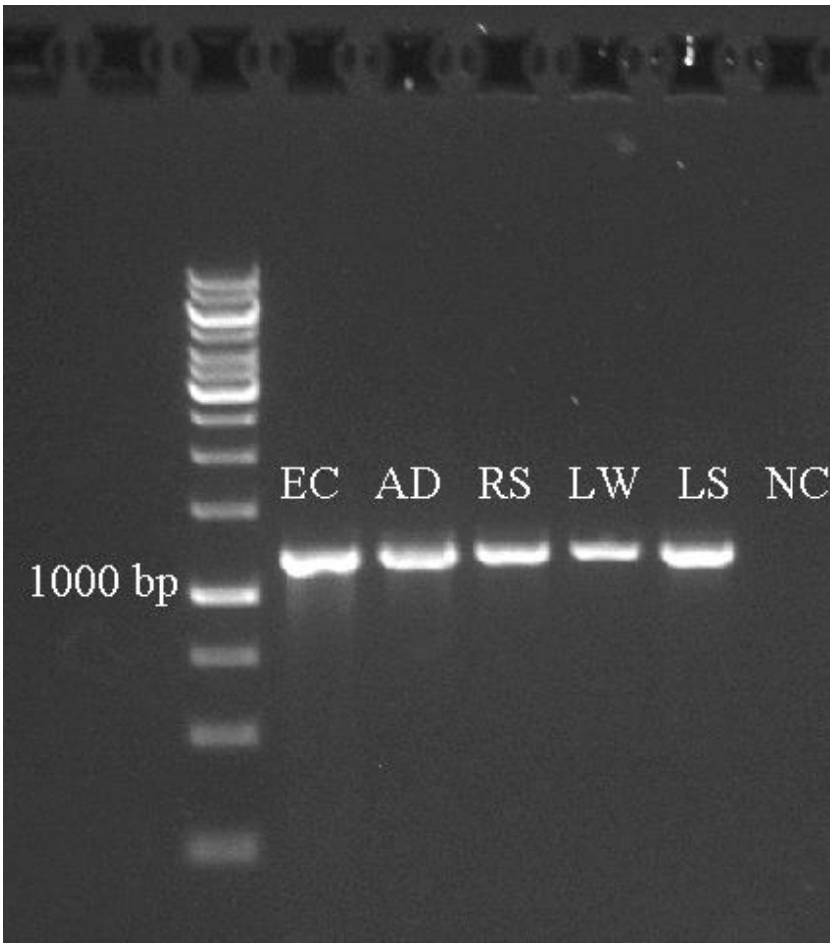

Figure S1 Gel image of the PCR products amplified by McrA169F and McrA1360R. EC: enrichment culture, AD: anaerobic digestion sample, RS: return sludge sample, LW: lake water sample, LS: lake sediment sample, NC: negative control.

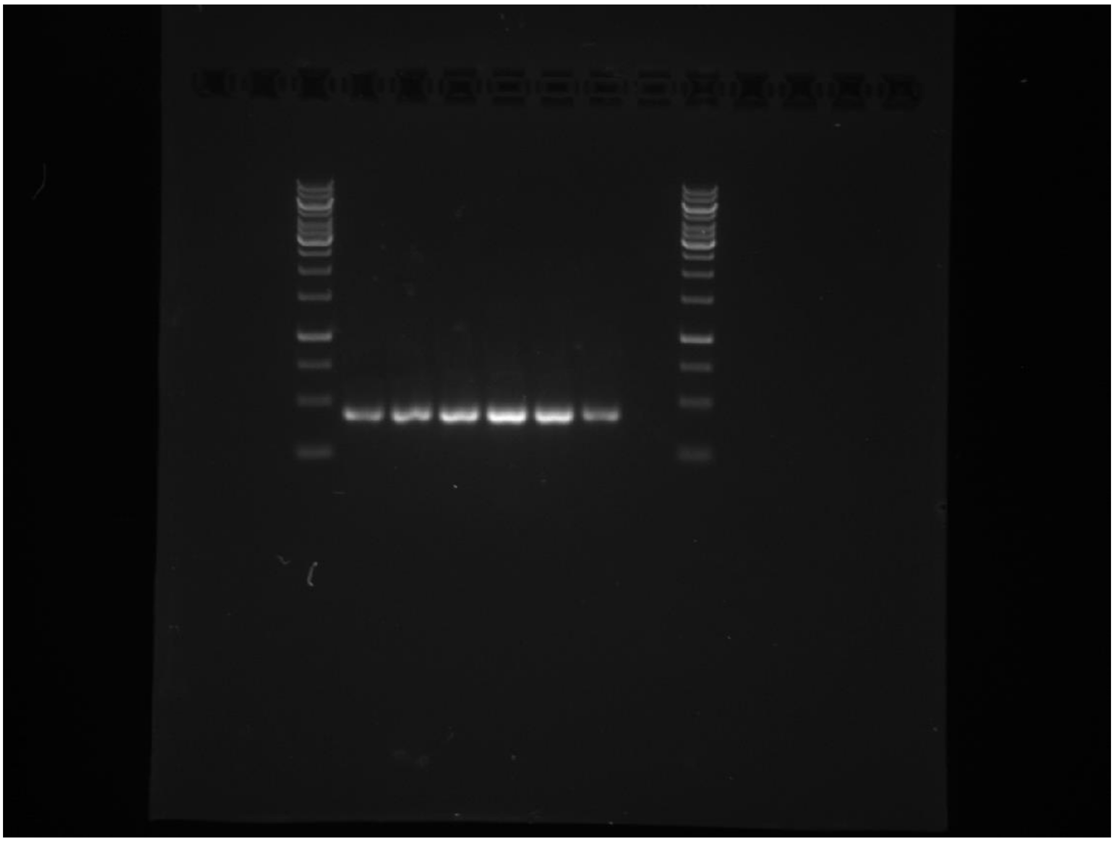

Figure S2 Original gel image of Figure 1a.

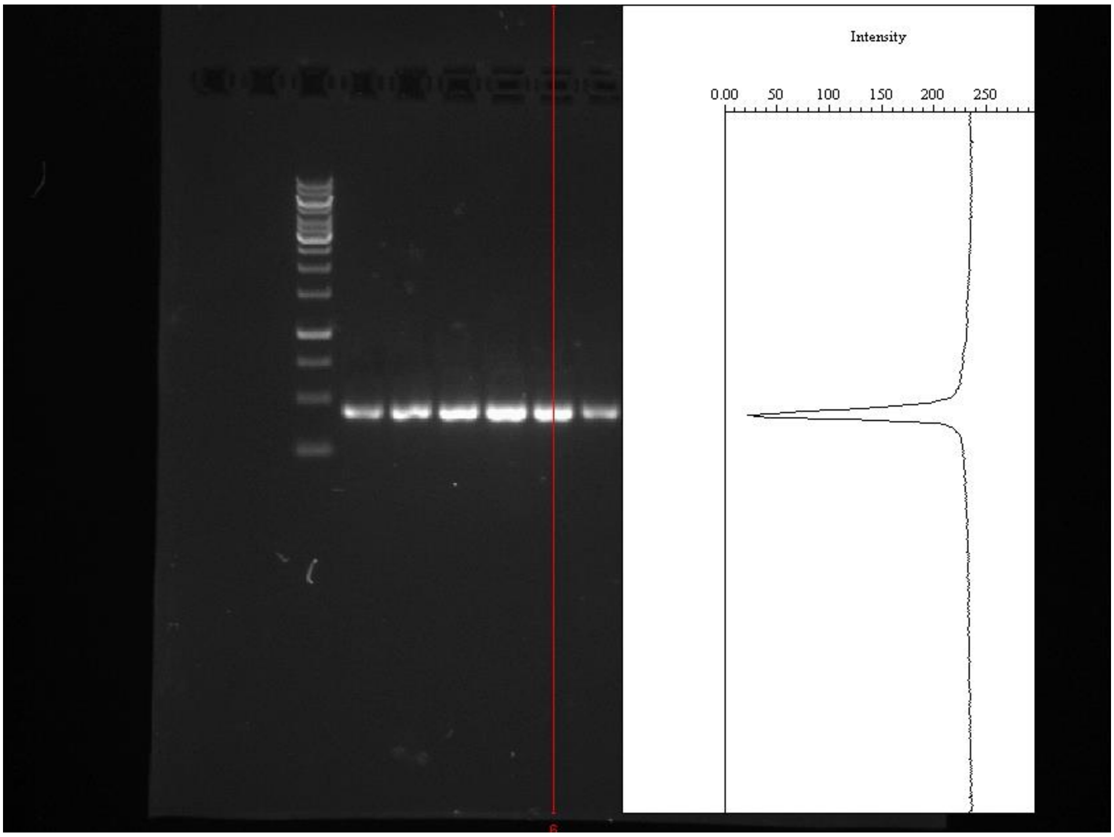

Figure S3 Band intensity analysis for Figure 1a.

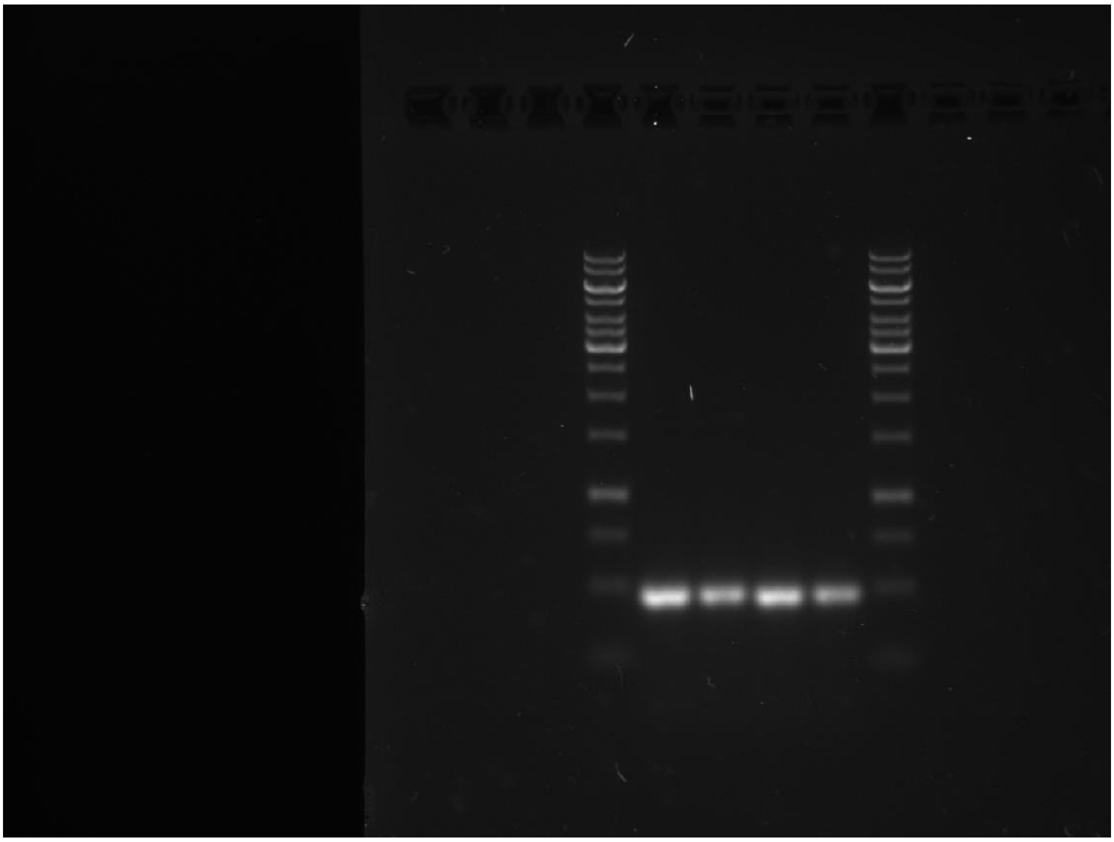

Figure S4 Original gel image of Figure 1b.

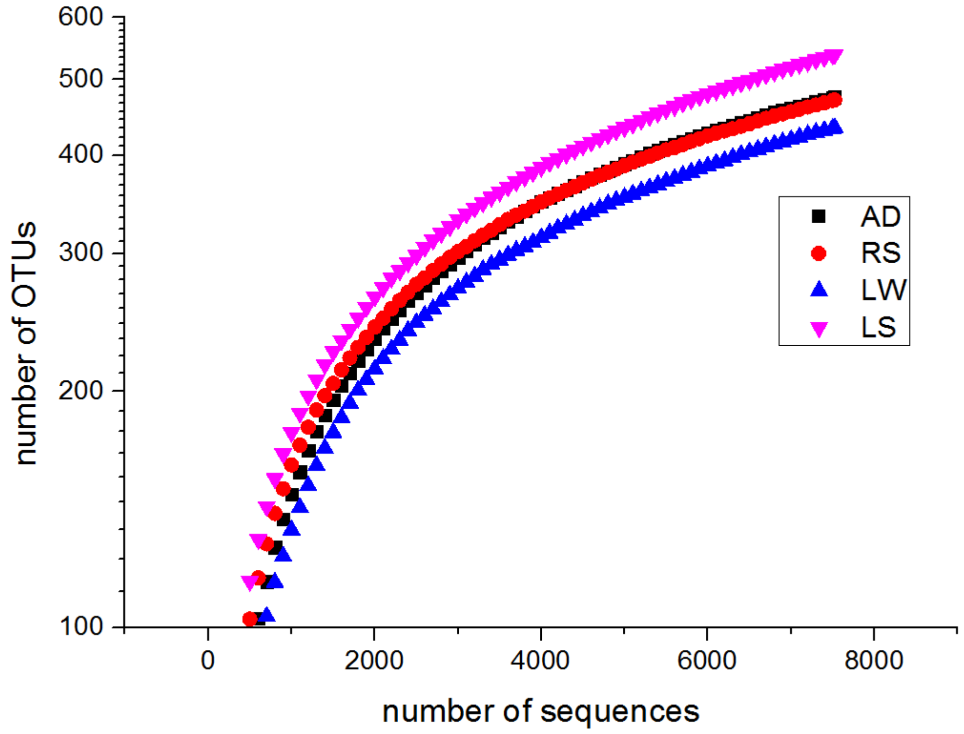

Figure S5 Rarefaction curves for OTUs (logarithmic scale) at the 0.03 cutoff level. AD: anaerobic digestion sample, RS: return sludge sample, LW: lake water sample, LS: lake sediment sample.

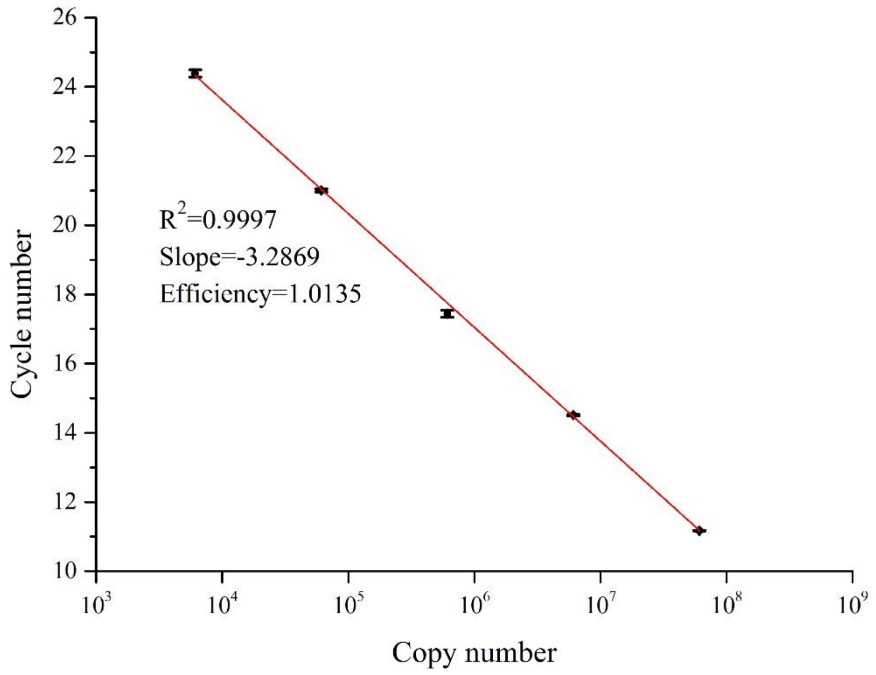

Figure S6 The calibration curve was generated from serial 10-fold dilutions of plasmid containing cloned *mcr*A gene fragments. Efficiency $=10^{-1/slope}-1$. The values were shown as average ± standard deviation based on three replicates.

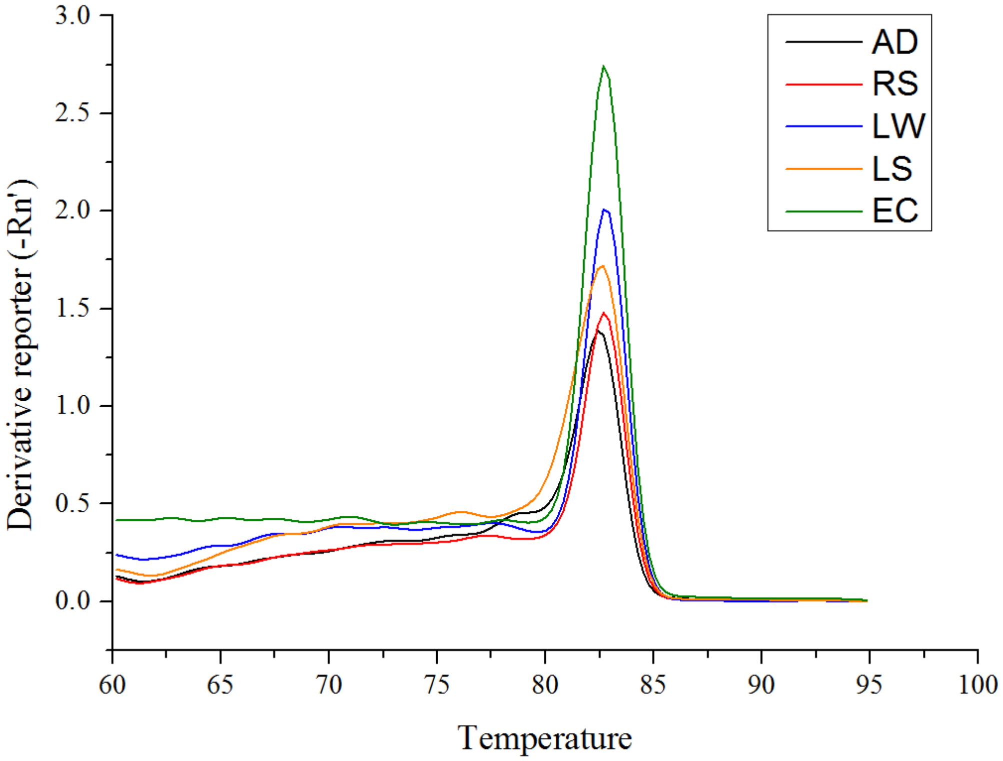

Figure S7 Melting curve analysis of the samples. AD: anaerobic digestion sample, RS: return sludge sample, LW: lake water sample, LS: lake sediment sample.

Table S1 Primers used in this study

Primers	Sequences (5' to 3')	Target
DP142F[1]	TAATACYGGATAGATCAAAG	
DP779R[1]	GCACCGCACCTGACACCT	16S rRNA gene of *M. nitroreducens*
DP569R[1]	GRACGCCTGACGATTRAG	
McrA169F[2]	GCAGCAATCACCAAGAAGAGAGG	
McrA1360R[2]	TGCCTCTTTGTGGAGGTACATGGA	*mcr*A gene of *M. nitroreducens*
McrA997F	ATCTGGCTCGGYGGCTACATGT	

Table S2 Similarity of the top 10 OTUs with *M. nitroreducens* genome sequences

OTU	Sequence similarity			
	Candidatus Methanoperedens nitroreducens (JMIY01000002)	*Candidatus* Methanoperedens MPEBLZ (LKCM01000102)	*Candidatus* Methanoperedens nitroreducens Vercelli (FZMP01000185)	*Candidatus* Methanoperedens BLZ2 (NTMG01000073)
OTU313	100%	83%	97%	83%
OTU679	99%	82%	96%	82%
OTU2596	99%	82%	96%	82%
OTU249	99%	83%	96%	83%
OTU515	99%	82%	96%	82%
OTU2891	99%	82%	96%	82%
OTU31	99%	82%	96%	82%
OTU50	99%	83%	96%	83%
OTU354	99%	82%	96%	82%
OTU35	99%	82%	96%	82%

Table S3 Basic information of the samples in this study

Sample	Sampling date	Sampling location	pH	NO_2^--N (mg/L)	NO_3^--N (mg/L)	NH_4^+-N (mg/L)	PO_4^--P (mg/L)
AD	28th June, 2017	27 °22' S/153 °08' E	7.8	ND[1]	0.23	692	134
RS	29th March, 2017	27 °22' S/153 °08' E	7.1	3.19	21.89	25.38	59.88
LW	24th, April, 2017	27 °29' S/153 °00' E	7.4	0.09	0.03	0.69	0.06
LS[2]	24th, April, 2017	27 °29' S/153 °00' E	7.1	0.23	0.25	112.69	0.23
EC	24th January, 2017	Laboratory	7.0-7.5	1.00	149.8	152.6	5.06

[1]ND: not detected

[2]nitrogen and phosphorus elements in the LS sample were extracted by KCl (2 mol/L) and the unit was mg/kg.

References:

1. Ding, J. *et al*. New primers for detecting and quantifying denitrifying anaerobic methane oxidation archaea in different ecological niches. *Appl Microbiol Biot*. **99**, 9805-9812 (2015).

2. Vaksmaa, A., Jetten, M. S. M., Ettwig, K. F. & Lüke, C. *Mcr*A primers for the detection and quantification of the anaerobic archaeal methanotroph '*Candidatus* Methanoperedens nitroreducens'. *Appl Microbiol Biot*. **101**, 1631-1641 (2017).
